# An analysis of patient reported maltreatment of distal radial fractures in Sweden

**DOI:** 10.1177/17531934251383111

**Published:** 2025-10-24

**Authors:** Ásgerdur Thórdardóttir, Marcus Sagerfors, Eva Lundqvist, Jonny K Andersson

**Affiliations:** 1 Department of Orthopedics and Hand Surgery, Faculty of Medicine and Health, Örebro University, Örebro, Sweden; 2 Atleva Gothenburg Handcenter and Department of Orthopedics, Institute of Clinical Sciences, The Sahlgrenska Academy, University of Gothenburg, Gothenburg, Sweden

**Keywords:** Complications, distal radial fractures, insurance claims, maltreatment, malunion, treatment cost

## Abstract

**Introduction:**

Distal radial fractures are the most common fractures in adults. Maltreatment of distal radial fractures can result in pain and disability. The most common complications and maltreatment after distal radial fractures are malunion, tendon injury, infection and nerve injury. The Swedish Patient Insurance Company (LÖF) indemnifies approximately 90% of all healthcare providers in Sweden. The aim of this study was to assess maltreated distal radius fractures using data from the LÖF-register in combination with data from the Swedish Fracture Register.

**Method:**

All insurance claims relating to patient-reported maltreatment of distal radial fractures in adults in Sweden, as well as data from the Swedish Fracture Register regarding fracture type and patient-reported outcome measures during the period 2011–2021 were analysed.

**Results:**

Of the total of 1393 claims relating to distal radial fractures during the study period, the frequency of reported maltreatment claims was 1.4%. Eighty-two per cent of the claimants were female and the median age was 60 years. Malunion was the most common overall complication, carpal tunnel syndrome was the most common nerve-related complication and extensor pollicis longus tendon rupture following anterior plating was the most common tendon-related complication. Sixty-four per cent of the claims were accepted and reimbursed. The total direct cost for maltreated distal radial fractures was €4,980,000.

**Conclusion:**

Reducing the number of symptomatic malunions, ensuring correct anterior plate placement and identifying other critical steps in treatment would likely improve patient safety and decrease the additional financial burden for the society.

Level of evidence: IV

## Introduction

Fractures of the distal radius are one of the most common fractures, with one national study showing an annual incidence of about 228 per 100 000 (Viberg et al., 2023). The treatment goal is to re-establish and maintain normal wrist anatomy to minimize future disability. Non-operative treatment in a plaster cast is successful in the majority (74%) of patients, but a few may require operative intervention mainly using anterior locking plate fixation (Rundgren et al., 2020; Viberg et al., 2023). Failure to treat appropriately, or complications of appropriate treatment can lead to loss of wrist motion and grip strength, as well as pain and difficulties with activities of daily living, which may lead to an overall increase in medical care costs, and an indirect cost to the society through increased insurance claims. The most common complications seen in distal radial fractures are malunion, tendon injury, infection and nerve injury (Katt et al., 2020; Rosenauer et al., 2020; Truntzer et al., 2018). Previous studies have addressed malpractice claims for fractures of the distal radius, but only in smaller groups of patients (Norum et al., 2018; Schweder et al., 2022).

The Swedish Patient Insurance Company (LÖF) indemnifies approximately 90% of all healthcare providers in Sweden. The total number of registered annual claims for all medical maltreatment in Sweden is more than 18,000, and approximately 40% are approved for compensation. Patients themselves must seek compensation for an avoidable injury, and LÖF collects information from the patient’s caregivers. All claims raised by patients are assessed by an independent expert physician within the specific medical speciality to determine whether the event was avoidable, and the extent of the permanent damage as well as medical disability based on the national guidelines and best clinical practise for treatment (Andersson et al., 2022; Om försäkringen, 2024). Claims submitted to LÖF are only eligible for compensation if the complication or adverse outcome is avoidable. Thus, poor outcomes from incorrect or delayed diagnosis and/or treatment, iatrogenic injuries or infections are covered, while known adverse outcomes from correctly prescribed treatments such as carpal tunnel syndrome associated with non-displaced fractures or rupture of the long extensor tendon to the thumb after non-surgical management are not. The injury is deemed to have been caused by maltreatment if the treatment provided has not adhered to the recommended national guidelines or best practice.

The primary aim of this study was to examine the causes of patient injuries resulting in distal radial fractures and to determine complications that require further medical care after maltreatment of distal radial fractures, by analysing patient injury claims filed with LÖF. The secondary aims were to assess fracture type and patient-reported outcome measures (PROMs) by linking the claims to data from the Swedish Fracture Register (SFR), and to analyse overall treatment costs as well as trends over time.

## Methods

All claims filed to LÖF related to distal radial fractures in adults (18 years of age and over) during 2011–2021 were extracted from their database in 2023, using the prespecified criterion of International Classification of Diseases Tenth Revision (ICD-10-SE) code S52.0–9. The search criteria were extended to include all types of wrist fractures and adjacent injuries, in order to reduce the risk of missing any distal radial fractures. Only claims related directly to distal radial fractures were included, and any claims relating to injuries other than distal radial fractures were excluded. All patient and fracture details were obtained from the documentation on the medical or claim forms completed by patients themselves. Informed consent was given by patients at the time of filing a claim with LÖF. The study was approved by the Swedish Ethical Review Authority (reference 2022-05471-01).

Data from claims that were accepted as well as ones that were declined, were assessed. Demographics such as age, gender, side of injury, and injury type were noted. Complications from treatment, missed or misdiagnosed fractures, and any secondary corrective surgeries were reviewed. The patients’ claims and medical records were examined, and the reasons for the claims were categorized by the first (AT) and senior (JKA) authors. In cases where the categorization was unclear, a consensus was reached after discussion with the second and third authors (EL, MS). Primary treatment was registered as operative treatment if the patient was operated on within the first 3 weeks of injury. Any further surgical procedures or initial surgical treatment after the first 3 weeks following injury was considered as secondary surgery. The accepted claims were divided into subgroups depending on the level of reimbursement and medical disability. The diagnosis of malunion of the fracture was obtained from the documentation on the medical records or the claim records often completed by patients themselves in their own words, with only written radiological medical reports available in the register. Patients often described the outcome in their own words such as pain, failure or loss of range of motion. Failure in osteosynthesis was defined as a failure to maintain the fracture position following internal fixation or implant-related problems such as penetration of a screw into the joint or soft tissue. Tendon ruptures and adhesions, as well as nerve injury and nerve compression secondary to treatment, were also documented when mentioned in the patient’s medical records.

Additional data relating to the patients and injuries were retrieved from the SFR. As of 2021, all orthopaedic units treating fractures of the distal radius in Sweden are affiliated with the Register. Fracture types based on AO/ASIF (Association of the Study of Internal fixation) fracture classification were extracted from the SFR register, along with the following PROMs: the EQ-5D, the EQ-VAS, the Arm Hand Function Index and the Bother Index of the Short Musculoskeletal Function Assessment. The PROMs noted in the SFR are collected at injury and a year later (Wennergren and Möller, 2018), thus allowing comparison of PROMs from the insurance patients with previously reported outcomes for distal radial fractures. Data in the SFR are linked to the patients’ social security number, which makes it possible to collect individual follow-up details.

The total compensation (including compensation for pain and suffering, scar, income loss, patient costs and disability) was extracted directly from the LÖF register and then calculated and summed up as direct costs. Indirect costs were not estimated, as it was not possible to retrieve the information on the length of the patients’ individual periods of sick leave. The Swedish Social Insurance Agency (Försäkringskassan) pays sick leave compensation to employed individuals, and the mean length of the sick leave period for ICD diagnosis S52.5 was 46.5 days.

## Statistics

Percentage and frequency were calculated and summarized for categorical variables. Median with range and mean with standard deviation were calculated for continuous variables. A simple linear regression was performed to detect trend over time for the number of claims. Numerical comparisons between two groups were performed with a Mann–Whitney *U*-test, and comparisons of categorical values with a chi-square test. Statistical significance was set to *p* < 0.05.

## Results

### Demographics

In total, 1393 patient claims for distal radial fractures were reported between 1 January 2011 and 31 December 2021. The total number of complications was 1637 as many patients had more than one adverse event per claim. Of these, 1139 (82%) claims were from females, and the left hand was affected in 751 patients (54%). Fifteen patients had bilateral injuries (1%). The median age was 60 years (range 18–94), and 933 were of working age (18–65 years). Claim applications were sent to LÖF at a median of 289 days (range 1–3631) after injury. A total of 888 (64%) compensation claims were approved.

**Figure 1.**
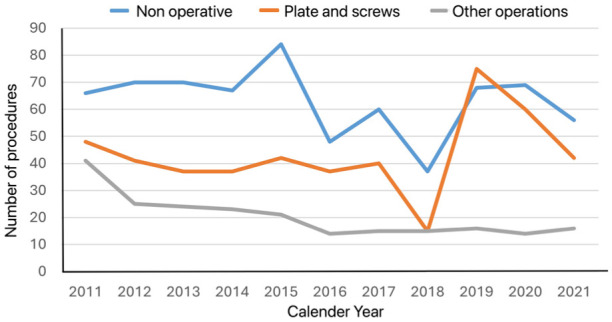
Trend over time for different treatment methods for distal radial fractures.

Causes of injury were low energy in 1127 (81%), high energy in 63 (5%), injury during sporting activity in 152 (11%) and unknown in 51 (4%). Six-hundred and ninety-eight patients (50%) received primary operative treatment within the first 3 weeks after injury. There were 18 patients (1%) whose injuries were initially missed and were untreated. The information was incorrect or missing in two patients. Of the 888 patients whose claims were reimbursed, 487 (55%) had primary operative treatment. There was an increasing trend in plate fixation as the primary treatment during the study period compared to other methods of operative intervention (Figure 1).

### LÖF

**Figure 2.**
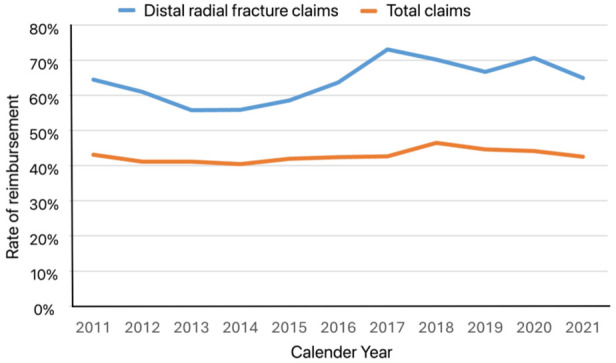
Rate of reimbursement of claims for distal radial fractures in comparison to the overall reimbursement rate for all claims.

**Figure 3.**
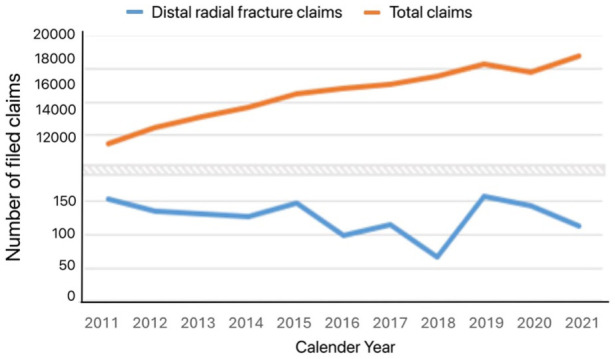
Trend over time for claims filed for distal radial fractures and total claims filed with LÖF.

The total number of claims filed with LÖF during 2011–2021 was 172,288, meaning that fractures of the distal radius represented just under 1%. However, 888 of the 1393 claims (64%) for distal radial fractures were compensated to some extent, which was significantly higher than LÖF’s overall compensation rate of 43% (*p* < 0.05). These trends were stable over the study period (Figure 2). No statistically significant change in the total number of claims filed for fractures of the distal radius was observed during the study period, although the total number of claims filed with LÖF increased approximately 6% every year (*p* < 0.05) (Figure 3). Among the 888 patients who received compensation, 658 patients (74%) were deemed to have some medical disability from an avoidable injury. The median medical disability caused by the injury, assessed by the expert physician at LÖF from standardized tables in patients with sequalae when the condition was stable at least 1 year after the incident, was 2% (range: 0–22%).

### Malunion

Malunion was the most common cause of all the claims (Table 1). Majority of these (70%) were accepted and reimbursed and the rest were deemed non-avoidable. The reimbursed patients had a median rate of one secondary operation (range 0–6); the total number of secondary operations in this group was 423.

**Table 1.** Type of complications with accepted and denied claims.

	*N*
Total claims	1393 (888)
Complications	1637 (1083)
Malunion	642 (448)
Non-operative treatment	504 (339)
Anterior locking plate	47 (36)
K-wire fixation	57 (45)
External fixator	28 (23)
Other treatment	6 (5)
Nerve related complaints	233 (170)
Carpal tunnel syndrome	132 (90)
Radial cutaneous nerve branch	44 (38)
Palmar cutaneous nerve branch	21 (17)
Iatrogenic median nerve laceration	7 (7)
Other nerve related complaint	29 (18)
Tendon related complaints	215 (146)
Extensor pollicis longus	114 (68)
Flexor pollicis longus	62 (46)
Other extensor tendon	22 (20)
Other flexor tendon problems	12 (10)
Not specified	5 (2)
Hardware failure	143 (126)
Screw penetration	64 (57)
Failure to maintain reduction	53 (47)
Other hardware complaints	26 (22)
Other causes of claims	404 (193)

Data provided as total number (accepted claims).

The total number of complications exceeds the total claims as some patients had multiple complications.

### Nerve-related complaints

Of all the 233 patients with nerve related complications, 61 were initially treated with an anterior locking plate and 53 non-operatively. The majority of these patients with nerve-related complaints received compensation (Table 1). Those that did not were mostly patients with carpal tunnel syndrome that were deemed non-avoidable. One-hundred and four of these patients had a carpal tunnel release, many of which were performed in combination with other procedures such as anterior plate extraction or a radial corrective osteotomy. Of the 44 patients with injury to the cutaneous branch of radial nerve, 23 were treated with either an external fixator or K-wires. All seven iatrogenic median nerve injuries were due to intraoperative nerve lacerations during insertion or extraction of an anterior locking plate.

### Tendon-related complaints

Extensor tendon injuries were much more commonly reported than flexor tendon complaints. Forty-four of 68 patients who had their claims accepted for extensor pollicis longus rupture were treated with anterior locking plates, with dorsal protruding screws being the main cause. All flexor pollicis longus (FPL) tendon injuries occurred following treatment with anterior locking plating. Those patients with FPL rupture who claims were denied were those who had correct placement of the anterior locking plate, and the rupture was deemed non-avoidable.

### Hardware issues

Intra articular penetration of screw or the dorsal prominence of tip of the screw contributed to most of the claims related to hardware. One-hundred and seventeen of these patients needed a further operative procedure at least once during the claim period.

### The Swedish Fracture Register

Of the 1393 LÖF claimants, only 444 were found to be linked to the SFR. These registered patients were demographically similar to the whole patient group regarding age, gender, treatment and degree of reimbursement. Out of these 444 patients, 171 had simple extraarticular (AO type A) fracture pattern, followed by complete intra-articular (AO type C) fractures in 107 patients and partial intraarticular (AO type B) fractures in 25. Those patients who responded to the PROMs questionnaire in this group were noted to have a statistically significant worsening of score at 1 year post injury (Table 2).

**Table 2.** Patient-related outcome measures for the patients in the Swedish Fracture Registry.

	Number of respondents (*n*)	Pre-injury	One year post-injury	*p-*Value
EQ-5D Index	142	0.897 (0.118)	0.812 (0.125)	**<0.001**
EQ-VAS	142	80.1 (22.6)	70.7 (21.8)	**<0.001**
SMFA Dysfunction Index	191	4.69 (11.5)	23.67 (20.5)	**<0.001**
SMFA Bother Index	179	9.47 (15.6)	28.42(20.8)	**<0.001**

Data shown as mean (standard deviation).

SMFA: Short Musculoskeletal Function Assessment.

Significant *p*-values shown in bold.

### Reimbursement Costs

The total reimbursement from LÖF to the patients was €4,980,000 (mean cost per patient €5600). The reimbursements were divided into compensation for pain and suffering (€1,719,850), scarring (€321,203), income loss (€518,087), patient costs (€75,658), disability (€2,234,234) and other costs (€110,968).

## Discussion

Despite fractures of the distal radius being the most common fracture seen in the emergency department (Rundgren et al., 2020; Schmidt et al., 2022), they account for less than 1% of annual claims to LÖF. Malunion seems to be the most commonly reported complication following distal radial fractures in Sweden, being responsible for just under half of the filed claims. This is in line with reports from other countries (DeNoble et al., 2014), but the findings are not directly comparable because of differences in the insurance systems between different countries in the Western world. As the true number of malunion and other complications after treatment of distal radial fractures in Sweden is unknown, it is difficult to estimate the proportion of patients with malunion or other complications from distal radial fracture who have not claimed compensation.

Our findings of carpal tunnel syndrome being the common nerve related complication from distal radial fractures, agrees with previous reports (Norum et al., 2018; Seigerman et al., 2019). Use of the Soong et al. (2011) classification for assessment of the prominence of volar locking plates in distal radial fracture surgery and for predicting the risk of flexor tendon complications was not possible in this cohort, as radiographs were unavailable. The incidence of FPL rupture was low in this study, which is similar to an earlier report (Floquet et al., 2021). One likely cause is underreporting of this complication. The rate of the claims in patients who were treated surgically in our series is higher than reported in an earlier study by DeNoble et al. (2014), and could possibly be due to increased use of anterior locking plates over time, which has been reported previously by other authors (Viberg et al., 2023).

Compared with a normal distal radial fracture population, patient data linked to the SRF in our study showed a relatively higher frequency of comminuted intraarticular fracture pattern (Sagerfors et al., 2023). This is to be expected as more complex fracture pattern is likely to experience more complications. The mean minimally important difference for the EQ-5D in our study group did not reach the required threshold of 0.1 (Rundgren et al., 2017), indicating that the observed decline may not be clinically relevant.

This study has several limitations. Although the number of patients with distal radial fracture patients being registered in the SFR is steadily increasing (Rundgren et al., 2020), during the period of this study not all orthopaedic departments in Sweden had begun reporting to the SFR. Hence, only a third of our study population were on the SFR database. The other main limitation is that these data include only those claims that have been reported by patients themselves. There is likely to be a group of patients with an unsatisfactory outcome from distal radial fracture during the study period who may not, for various reasons, have submitted a claim. Therefore, the true number of patients with an adverse outcome following distal radial fractures who require further medical care after maltreatment of distal radial fractures will be unknown. Moreover, we did not have access to the patients’ radiographs directly, but only the written reports, and hence were unable to assess the degree of malunion ourselves. However, in people over 60 years of age, it has been shown there is little correlation between radiographic outcome and the pain and function (Lawson, 2023). Therefore, it could be argued that what is probably more important is the true number of patients with residual symptoms from a malunion rather than the degree of deformity itself, and little more is gained by getting additional information on the extent of malunion.

Malunion appears to be the most common factor leading to an unsatisfactory outcome following a distal radial fracture. The increasing trend in treating these injuries primarily using an anterior locking plate does not seem to have eliminated this risk. The potential problems relating to maintaining fracture reduction and implant related issues remain as a cause of maltreatment claims. However, these risks, along with iatrogenic nerve and tendon related problems, could partly be influenced with meticulous surgical techniques focussing on tissue handling, fracture reduction, correct implant placement and screw length measurement. These could have a substantial impact on future maltreatment claims, reducing the overall treatment costs and the burden it puts on the society in general.
